# Photocatalytic degradation of oily waste and phenol from a local South Africa oil refinery wastewater using response methodology

**DOI:** 10.1038/s41598-020-65480-5

**Published:** 2020-06-01

**Authors:** E. K. Tetteh, S. Rathilal, D. B. Naidoo

**Affiliations:** Faculty of Engineering and the Built Environment, Department of Chemical Engineering, Durban University of Technology, Steve Biko Campus Block S4 Level 1, Box 1334, Durban, 4000 South Africa

**Keywords:** Environmental impact, Environmental, health and safety issues, Chemical engineering, Chemical hydrogen storage

## Abstract

The photocatalytic degradation of a local South Africa oil refinery wastewater was conducted under UV radiation using an aqueous catalyst of titanium dioxide (TiO2), Degussa P25 (80% anatase, 20% rutile) in suspension. The experiment was carried out in a batch aerated photocatalytic reactor based on a central composite design (CCD) and analyzed using response surface methodology (RSM). The effects of three operational variables viz. TiO_2_ dosage (2–8 g/L), runtime (30–90 minutes), and airflow rate (0.768–1.48 L/min) were examined for the removal of phenol and soap oil and grease (SOG). The data derived from the CCD, and the successive analysis of variance (ANOVA) showed the TiO_2_ dosage to be the most influential factor, while the other factors were also significant (P < 0.0001). Also, the ANOVA test revealed the second-order of TiO_2_ dosage and runtime as the main interaction factors on the removal efficiency. To maximize the pollutant removal, the optimum conditions were found at runtime of 90 minutes, TiO_2_ dosage of 8 g/L, and an aeration flow rate of 1.225 L/min. Under the conditions stated, the percentage removal of phenol (300 ± 7) and SOG (4000 ± 23) were 76% and 88% respectively. At 95% confidence level, the predicted models developed results were in reasonable agreement with that of the experimental data, which confirms the adaptability of the models. The first-order kinetic constants were estimated as 0.136 min^−1^ and 0.083 min^−1^ for SOG and phenol respectively.

## Introduction

Photocatalysis has attracted worldwide interest due to its potential to use solar energy not only to solve environmental problems but also to provide renewable and sustainable energy^1,2^. However, the ever-increasing demand for good water quality and oil refinery products have become expensive commodities with major challenges, which requires thoughtful attention^3,4^. Oily waste from petroleum refinery has aliphatic and phenolic compounds, are considerably carcinogenic and toxic to the ecosystem and human health, and therefore their removal from wastewater is of global concern^3,5,6^. The tox,icity and extreme instability of oily waste make them not degradable easily, this pose a significant threat to the water bodies and soil^5–7^. Meanwhile, the processing of crude oil consumes large volumes of water and generates about 0.4–1.6 times the amount of crude oil processed as oil refinery wastewater (ORW)^8–10^. The discharge of ORW with adequate or no treatment, due to their inertness, endocrine-disrupting abilities, and carcinogenic behaviour could also affect water bodies and soil profile negatively^9–11^. Therefore, there is the need to develop sustainable and eco-effective methods to mitigate the oily pollutants from the ORW, to produce clean water and a sustainable environment^12^. In this case, the photocatalysis technology was considered.

Unfortunately, the production of polluted wastewater (ORW) is based on the raw crude oil type, plant configuration, and operational procedure, which varies in physicochemical parameters as compared to urban wastewater^13,14^. ORW may constitute a high range of hydrocarbons in free, dispersed and dissolved forms, like SOG (600–1500 mg/L), phenol (20–300 mg/L), chemical oxygen demand (200–950 mg/L) and other pollutants^10–13^. The rapid increase of emerging contaminants (such as oily waste, pharmaceuticals, endocrine-disrupting drugs, antibiotics, and pesticides) in recent times has raised concern with respect to upsetting the general balance in the ecosystems and the wastewater settings^12–14^. Conventionally, these emerging contaminants cannot be completely degraded by a standalone chemical, mechanical, or biological treatment without advanced technology^14,15^. To combat this crisis, the policy of stringent environmental protection has become an indubitable principle in various developing countries^13–16^. South Africa, which cannot be exempted from the ORW environmental threats, has admissible limits of 50 mg/L and 5 mg/L for carcinogenic contaminants like SOG and phenol respectively^16,17^.

To meet some of the aforementioned stringent bylaws^16^, physicochemical and mechanical techniques such as hydrocyclones, plate separators, and flotation is commonly used as a pre-treatment process of ORW^18–20^. Usually, these techniques work by gravity separation and are not effective without the application of coagulation which then increases the cost of chemical usage^21–23^. In this regard, several technologies have been proposed including; adsorption, biological systems, membranes, microwave-assisted catalytic wet air oxidation (hydrothermal oxidation), and advanced oxidation processes (AOPs)^22–27^. Unfortunately, some of these technologies are associated with many drawbacks including residual sludge generation, low efficiency, reaction rate, and operational conditions control within a specified temperature and pH for the treatment of ORW^28–30^.

AOP in recent times is gaining attention for mineralization of ORW because it offers distinct merits over many conventional treatments^31^. In comparison, AOP has the potential to destroy a wide range of organic and recalcitrant pollutants to completion within a shorter possible time than biological systems^32–34^. Classifying types of AOPs are generally based on the techniques used in generating the reactive radicals and the relative oxidation potential (estimated to be +2.8 V). Some of the oxidants like ozone, H_2_O_2_, HOCl, and chlorine have high oxidation potentials as 2.07 V, 1.78 V, 1.49 V, and 1.36 V respectively^31–36^. The single-use or combination of any of these oxidants exposed to ultraviolet (UV) or sunlight radiation produces oxidative species (OH^−^, O^2−^)^36,37^. Hydrogen peroxide (H_2_O_2_), for instance, can hasten the reaction rate, capture electrons, and react with excess oxygen by absorbing the light with a shorter wavelength less than 310 nm, which makes it energy-intensive^36–38^. On the other hand, decomposition of an electron-hole charge pair is formed when semiconductors like ZnO; WO_3_ or TiO_2_ are illuminated with UV light^38–40^. In all the cited scenarios, the generation of OH radicals is very essential for the photocatalysis process due to their high reactivity^39–41^. Among them, titanium dioxide (TiO_2_), has been the most effective photocatalyst or semiconductor for wastewater applications^31^.

Heterogeneous photocatalytic oxidation (HPO) that utilizes TiO_2_, when exposed to UV light is said to produce the most powerful intermediary oxidative radicals. HPO is seen as a promising route for degrading recalcitrant organic pollutants with less toxic substances^31–34^. As most promising technologies are associated with complex operating parameters that can affect their performance, HPO cannot be left out^37,41,42^. Some of these variables include light intensity, amount of catalyst, pH, temperature, the concentration of the pollutants, aeration flowrate, wavelength, and reaction time^34–38^. A solar photocatalytic degradation was conducted on TiO_2_ in an immobilized system; it was found that increasing the catalyst dosage from 0.5 to 5 g/L, resulted in chemical oxygen demand (COD) removal of 55 to 83%^39^. However, the use one-factor-at a time (OFAT) to investigate multi-function operating systems have limited information on the overall interactive effects of the factors on the response^43,44^. Addition to this, the OFAT approach which requires a lot of resources is time-consuming and cost-intensive^44,45^.

Response surface methodology (RSM) has been referred as the alternative to the OFAT approach for optimization, and has also shown the overall interactive effects of factors in numerous chemical and wastewater treatment processes^17,43–45^. RSM is a collection of statistical and mathematical techniques used for experimental design and process optimization as well as improving existing process design^17,45^. However, to the best of our knowledge, little is known about using RSM to optimize the photocatalytic degradation of ORW. Thus to specify the most influential and interactive factors in order to enhance the system efficiency. Therefore, in this work, the central composite design (CCD) was employed to model and optimize the degradation of local South Africa ORW using TiO_2_ and UV light. The factors considered were TiO_2_ dosage, runtime, and aeration flow rate, whereas the removal of SOG and phenol were the responses.

## Materials and Methods

### Oil refinery wastewater sample

A local South Africa ORW sample from a point source leaving a dissolved air flotation to the clarifier into the sewer was collected, characterized, and monitored for its water quality within a period of four months. A synthetic sample was then prepared according to Naidoo^46^, to mimic the raw ORW for the laboratory test. This was prepared by adding 3 mg/L phenol crystals and 40 mg/L Power Glide SAE40 motor vehicle oil (Engen, SA) to 1 L of ORW. The oil-water emulsion obtained was stirred for 24 hours and allowed to stand for 2 hours, ensuring there was no non-dispersed oil in the water. The supernatant was filtered using a quantitative filter paper (Whatman grade 597) with a pore size of 55 mm. American Public Health Association standard method of wastewater analysis was followed to characterize the synthetic ORW as depicted in Table 1. The phenol was tested with a ThermoFisher Gallery Discrete Analyser (manufactured by ThermoFisher Scientific). The SOG was measured according to a modified method by Tetteh *et al*.^10^. The mineralization of the SOG and Phenol was the key responsibilities for this study. Three samples were taken and the degradation average values were used for the RSM. The degradation percent for the SOG and Phenol after each experimental run was calculated using Eq. (1).

1 $$ \% \,degradation=\frac{{C}_{0}-C}{{C}_{0}}\times 100 \% $$

**Table 1:** Characterised oil refinery wastewater sample before and after treatment.

Component	Before value ± std dev	Treated value ± std dev
SOG (mg/L)	4000 ± 23	480 ± 36
Phenol (mg/L)	300 ± 72	69 ± 15
Phosphate (mg/L)	60 ± 33	35 ± 14
Calcium hardness (mg/L)	37 ± 24	9.25 ± 32
M-Alkalinity (mg/L)	77 ± 32	19 ± 4
Total Dissolved Solids (TDS) (mg/L)	233 ± 53	82 ± 76
pH	7.13 ± 12	7.8 ± 1
Iron (mg/L)	6 ± 13	3.3 ± 45
Chlorides (mg/L)	99 ± 22	34.65 ± 23
Sulfates (mg/L)	28 ± 23	12.6 ± 1
Silica (mg/L)	60 ± 23	15 ± 32

In this equation, *C*_0_ and *C* are the initial and remaining concentrations respectively.

### Titanium dioxide (TiO_2_)

The Degussa P25 purchased from Huntsman Tioxide South Africa (Pty) is the TiO_2_ catalyst used. This is made up of anatase (80%) and rutile (20%) with a mean particle size of 30 nm and a surface area of 50 m^2^/g. The physicochemical properties of this nanocatalyst are given in Table 2. A stock suspension of 1 L TiO_2_ nanoparticle was prepared and stored at 21 °C. To ensure homogeneous mixing, the TiO_2_ suspension was sonicated at 20 kHz for 15 min using VWR model 75HT sonicator (VWR, Mississauga, ON, Canada). The pure TiO_2_ nanoparticle was characterized using SHIMADZU x-ray diffraction (XRD) (Model: XRD 6000) coupled with Cu- K radiation at λ = 0.15418 nm, a voltage of 20 kV and a current density of 30 mA. This was carried out at room temperature, whereas the mean crystallite size (D) was estimated using the Scherrer formula (2)^47,48^.

2 $$D=\frac{k{\rm{\lambda }}}{\beta \,\cos \,\theta }$$

where, the X-ray radiation wavelength, the Scherer constant, the maximum peak full width at half radians, and the Bragg diffraction angle (radians) are represented by λ = 0.15418, k = 0.89, β and θ respectively.

**Table 2:** Physicochemical properties of titanium dioxide (Degussa P25).

properties	value
White powder content	94% purity
Phase mixture	Rutile 20%, Anatase 80%
Surface gravity	4.1 g/cm^3^
Crystal size	0.23 m
Bulk density	1.1 g/cm^3^
Oil adsorption	18 cm^3^/100 g pigment
Durability	High durable
Mean particle size (nm)	30
pH in aquatic solution	5.5
ISO 591 classification	R2
CAS No	13463-67-7

### Experimental procedure

**Figure 1:**
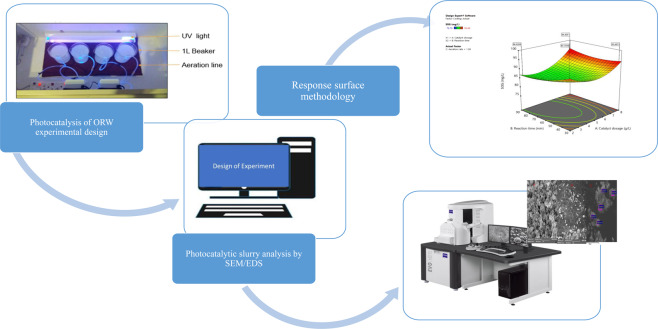
Schematic of Photocatalysis experiment.

The photocatalytic reaction was performed in a modified glass reactor chamber with four beakers of 1 Litre volume each, stirred at 150 rpm using a magnetic stirrer. In previous studies^10,46^, oily waste treatment was found to be more effective in the acidic medium than alkaline medium, hence in this study, the pH of the sample was kept constant at 5.5. The adjustment of the pH was done with sulfuric acid and sodium hydroxide solutions. Two lamps of fluorescent T8 blacklight- blue bulb (18 W, Philips, and Netherland), were suspended above the beakers to ignite the photons of the TiO_2_ catalyst for the degradation of the pollutants. To achieve the full UV light intensity (200–430 nm), before the experiment the lamps were turned on for at least 30 min. A DARO Twin aquarium air pump with a double outlet, high and low flow setting, pinholes (0.5 cm), and length, 9 cm was used as the aeration source. After each set time of runs, samples were collected using a syringe and filtered through a 0.22 μm nylon syringe filter for analysis. The residue was characterized by a Scanning Electron Microscope (SEM) coupled with an energy dispersive x-ray analyzer (EDX) (Model: EVO HD15, Carl Zeiss, Germany) as shown in Fig. 1.

### Experimental design using RSM

Design-Expert software (Version 11.1.0.1, Stat Ease Inc, USA) was used for the multivariate regression analysis, modeling, and optimization via the RSM-CCD^45^. The three selected factors with their respective levels were denoted by A, B, and C for catalyst dosage (2, 5, 8 g/L), runtime (30, 60, 90 min), and airflow rate (0.77, 1.11, 1.48 L/min). The input variables lower and upper limits were specified together with SOG and Phenol removal as the response variables to generate the experimental matrix. By applying the CCD, 20 experimental runs were designed with 6 center points, 8 factorial points, and 6 axial center points. The data obtained (Table 3) were then fitted on a quadratic polynomial model to analyze the relationship between the response variables and the input factors using (3).

3 $${\rm{Y}}={\beta }_{0}+\mathop{\sum }\limits_{i=1}^{k}\,{{\rm{\beta }}}_{{\rm{i}}}{x}_{i}+\mathop{\sum }\limits_{i=1}^{k}\,{{\rm{\beta }}}_{{\rm{ii}}}{x}_{i}^{2}+\mathop{\sum }\limits_{1\le i\le j}^{k}\,{{\rm{\beta }}}_{{\rm{ij}}}{x}_{i}{x}_{j}+\varepsilon $$

**Table 3:** The CCD input and output data obtained.

Run	Factor 1	Factor 2	Factor 3	Y1-SOG (%)		Y2-Phenol (%)	
	A: Catalyst dosage (g/L)	B: Run Time (min)	C: Air Flow rate (L/min)	Exp	Pred	Exp	Pred
1	2	60	1.11	87.21	88.27	47.98	47.59
2	8	30	1.48	87.21	86.3	38.39	39.86
3	5	60	1.11	85.71	85.61	49.6	47.46
4	2	90	0.768	79.73	80.57	56.58	55.49
5	2	30	1.48	85.71	85.57	34	32.74
6	5	60	0.768	79.73	79.19	55.97	54.15
7	8	90	1.48	85.71	86.63	64.92	63.54
8	5	60	1.48	76.79	77.63	42.71	42.92
9	8	60	1.11	93.69	92.94	49.6	48.38
10	5	60	1.11	85.71	85.61	53.86	47.46
11	8	90	0.768	89.04	89.15	49.6	51.26
12	5	60	1.11	85.71	85.61	33.52	47.46
13	5	90	1.11	87.21	86.06	49.6	49.45
14	5	60	1.11	85.71	85.61	45.33	47.46
15	2	90	1.48	79.73	79.01	33.52	34.48
16	5	60	1.11	85.71	85.61	49.6	47.46
17	5	30	1.11	87.21	88.67	45.33	43.87
18	8	30	0.768	87.21	87.85	41.85	41.31
19	2	30	0.768	87.21	86.17	65.69	67.48
20	5	60	1.11	85.71	85.61	49.6	47.46

In the above equation, *Y* denotes the response variable of the photocatalytic efficiency, *β*_0_ is a constant, *β*_*ij*_, *β*_*ii*_, *β*_*i*_ are the coefficients of regression for interaction effects, *x*_*i*_, *x*_*j*_ are independent variables and *ε* represents the error.

## Results and discussion

### Characterization of TiO_2_

**Figure 2:**
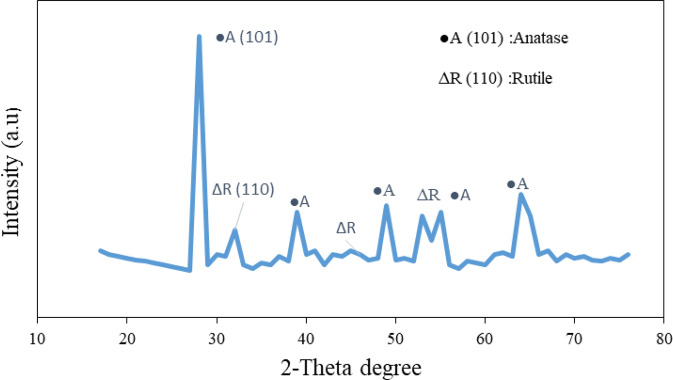
XRD pattern for TiO_2_ (Degussa P25) with reference line patterns assenting JCPDS: No 21-1272 to ∙ A (101): Anatase and No 21-1276 to ∆R (110): Rutile crystalline.

Figure 2 shows the XRD spectrum of the TiO_2_ (commercialized Degussa P25) nanoparticles with distinct diffraction peaks of anatase (101) and rutile (110) nanoparticles^47,48^. The diffraction peaks correspond to a reference line pattern (JCPDS: 21-1272/76) of crystalline titanium dioxide^48^. This confirms that Degussa P25 has a crystalline structure, which consists of anatase and rutile phases. The 2θ at peaks 28.35°; 37.78°; 54.98° and 65° confirm the strong diffraction peaks of anatase (A) phase, whereas peaks at 32.3°; 47.5°; 48.1^o^ and 53.95^o^ can be attributed to rutile (R) phase^46–48^. As anatase (101) is preferentially oriented with more hydroxyl groups at its reactive surface with less capacity of oxygen, makes it suitable to absorb the SOG and phenol compounds present in the ORW^46,47,49^. The average grain size of the nanoparticles was estimated by using the x-ray line augmentation technique together with the Scherrer (2)^48–50^. The estimated size for the anatase (110) and rutile (101) respectively were about 23 nm and 39 nm, which was close to what has been reported^50,51^. The estimated particle size and crystal structure signify scattering rising from large crystals produce Bragg peaks as a function of the reflection angle^48–51^.

**Figure 3:**
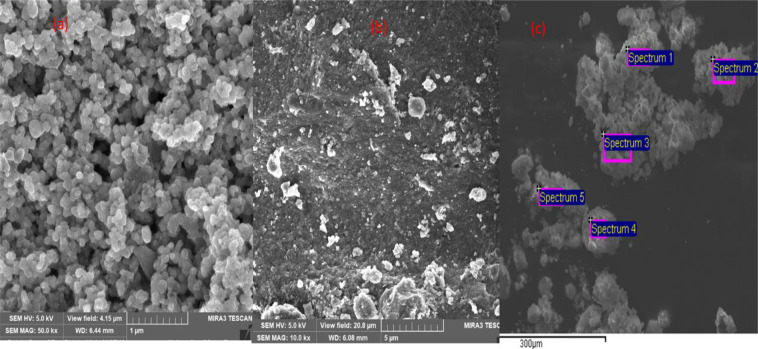
SEM image of TiO2 (Degussa P25) photocatalyst (**a**) Pure form (**b**) Slurry form and (**c**) slurry in EDS image.

Figure 3 presents the SEM image (a and b) of the TiO_2_ nanoparticle morphology before and after the experiment, as well as the EDS image (c). Two different grain sizes among the agglomerated particles with uniform spherical shape are observed in the SEM image (a and b). Their surface layers were seen to be heterogeneous and porous with a thickness about 5–10 µm^48,50^. This denotes a good inter-particle bond that was formed between the anatase and rutile particles (Fig. 2) with high porosity which enhanced the degradation whilst in suspension^47^. This confirms the measurement obtained from the XRD pattern (Fig. 2), where the anatase phase is predominant^48^. Likewise, the estimated smaller and larger particle size appears to be about 23 and 39 nm respectively^47,48^. This suggest that the smaller particles are the anatase whereas the larger particles are rutile as established by other authors^47–51^.

**Figure 4:**
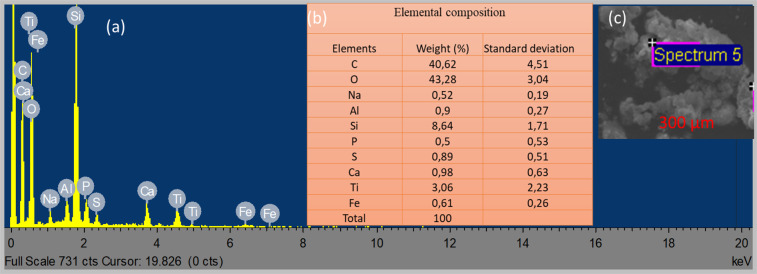
EDS image of TiO2 (Degussa P25) Photocatalysis slurry; (**a**) Peaks; (**b**) Elemental distribution; (**c**) Spectrum.

Figure 4 shows the EDS peaks (a) with its corresponding elemental distribution (b) deposited on the photocatalyst slurry. The visible pores (spectrums 5) on the surface layer (Fig. 3c) signifies agglomeration occurrence during mineralization^50,51^. From the EDS image (a- peaks; b-table), it was certain the chemical composition in the wastewater can suppress the TiO_2_ photocatalyst^51,52^. The appreciable increasing order of compounds presence were as follows: Oxygen (O) > Carbon (C) > Silica (Si) >Titanium (Ti) > Calcium (Ca) > Aluminum (Al) > Sulphur (S) > Iron (Fe) > Sodium (Na) > Phosphorus (P). Also, the broad diffraction peak at 2θ = 15–25° in the XRD (Fig. 1) might also be the amorphous silica layer^48,51^. This is likely to rapture an amount of the TiO_2_ photocatalyst reactive site from oxidation and might suppress the photocatalytic efficiency^48,50,52^. Moreover, chains of octahedral, aromatic, and aliphatic species (C-C/C-H; C-O-H/C-O-C; C=O; O-C=O) are able to bridge with TiO_2_ in edge-sharing configurations^50–52^. As it appears in the EDS image (Fig. 2a), with a high amount of oxygen (O) and carbon (C) denotes the higher possibility of passive layer formation^52,53^. Such as carbon-oxygen –contamination nano-layer (C-O-N/SOG), which therefore enhanced the removal of the SOG and phenol^52–54^. The SEM/EDS and XRD analysis confirmed the unique hydrophobic features of TiO_2_ (Degussa P25) nanoparticle^50,51,54^. These include high surface area (50 m^2^/g), particle size (mean size 30 nm), multifunctional phase (rutile and anatase) and photocatalytic degradation ability giving it a great potential for water and wastewater treatment^50–54^.

### Response surface methodology

The use of the OFAT approach on the photocatalytic system has been extensively reported by many authors^48–50^. However, the interaction effects of multiple factors on the response variables is still a limitation^46,47^. Therefore, the relationship between three factors (catalyst dosage, reaction time, and aeration flow rate) on the response variables (SOG and Phenol) were then examined using RSM. For graphical representation, three-dimensional response surface plots were used to ease visualization of the interaction effects of the input factors^46,55,56^. The experimental data obtained for the removal of SOG and phenol using the photocatalytic system with TiO_2_ nanoparticle is presented in Table 3. The data obtained were then fitted onto a reduced quadratic model using the Design-Expert software (Version 11.1.0.1, Stat Ease Inc, USA) model techniques^46^. A good correlation was observed, with a predicted value slightly deviated by less than 5% from the experimental data^46^. The high level of the TiO_2_ catalyst dosage increased the mineralization level of SOG and Phenol^53,54^, such that the release of more of the active H^+^ and OH^−^ radicals enhanced the degradability of the contaminants by absorbing them unto the TiO_2_ active surface^50–52^.

#### Model fitting and statistical analysis

Experimental data obtained was modeled unto a second-order polynomial function (quadratic) model. The models were developed to relate the response (Y_1_ and Y_2_) as a function of the coded input factors (A, B, and C). Akaike’s Information Criterion (AlCc) with the forward direction was used as a criterion to select the best model. The improved models are expressed in Eqs. (4) and (5) for SOG and Phenol removal respectively.

4 $$\begin{array}{ccc}{Y}_{1}({\rm{SOG}}) & = & 85.59+2.33{\rm{A}}-1.31{\rm{B}}-0.777{\rm{C}}+1.72{\rm{AB}}-0.2377{\rm{AC}}\\  &  & -0.2416{\rm{BC}}+4.99{A}^{2}+1.75{B}^{2}-7.18{C}^{2}\end{array}$$

5 $$\begin{array}{ccc}{Y}_{2}({\rm{Phenol}}) & = & 47.23+0.7245{\rm{A}}+2.92{\rm{B}}-5.62{\rm{C}}+5.48{\rm{AB}}+8.32{\rm{AC}}+3.43{\rm{BC}}\\  &  & +0.53{A}^{2}-0.795{B}^{2}+1.3{C}^{2}\end{array}$$

The model-independent terms (A, B, and C), interaction terms (AB, AC, and BC) and the quadratic terms (A^2^, B^2^, and C^2^) were found to be significant (P < 0.0001). These were selected based on the P-values being less than 0.05 and adequate precisions being greater than 4 with the regression coefficient closer to 1^55,56^. The negative and positive coefficient terms show that individual or interaction terms in the model can affect the response either decreasing or increasing the degradation efficiency^45^. Thus, the positive and negative coefficient term(s) significantly influence the photocatalyst process^38,40^. The trend in decreasing the removal efficiency of SOG removal, in relation to decreasing of the model (4) terms are as follow A^2^ < A < B^2^ < AB < AC < BC < C < B < C^2^. Likewise, the decreasing order of the phenol model (5) terms is AC < AB < BC < B < C^2^ < A < A^2^ < B^2^ < C. It is deduced that the interaction effects of catalyst dosage and airflow rate (AC) or catalyst dosage and runtime (AB) can enhance the degradation, whereas the most sensitive factor among them is the catalyst dosage (A). This might be due to sufficient active surface area available for adsorption of the contaminants^38,44^.

Tables 4 and 5 present the regression analysis of variance (ANOVA) of the second-order polynomial models examined for the SOG (4) and Phenol (5) removal respectively. ANOVA results constitute source (the source of the variation), DF (degree of freedom), the sum of squares, mean squares, Fisher (*F*-values), and Probability (*P-*values). The *F*-test is used to determine the significance of the regression coefficients of the parameters. The low coefficient of variation (CV) values of 1.21% and 10.95% respectively for the SOG and phenol removal confirms the accuracy of the models^55^. The models had an adequate signal-to-noise ratio (adequate precision ratio) of 10.97 and 9.37 for SOG and phenol removal respectively^45^. The P-values <0.05 confirms the significance of the models. Also, the model’s predictability was also confirmed by the lack of fit of the models, which are not significant relative to the net error, such that their p-values are greater than 0.05^45^. The adequate precision value is greater than 4, which signifies the models can be used to navigate the design space^44^. The diagnostic plots (in Supplementary Fig. S1) were also used to examine the residual analysis of the response surface, which proves the data obtained were well fitted on the models^43–45^.

**Table 4:** Analysis of Variance (ANOVA) for SOG response surface.

Source	Sum of Squares	df	Mean Square	F-value	p-value	significant
Model	262.33	9	29.15	27.4	<0.0001	
A-Catalyst dosage	54.06	1	54.06	50.81	<0.0001	
B-Run Time	17.29	1	17.29	16.25	0.0024	
C-Air Flow rate L/min	6.04	1	6.04	5.67	0.0385	
AB	23.77	1	23.77	22.34	0.0008	
AC	0.452	1	0.452	0.4248	0.0292	
BC	0.467	1	0.467	0.439	0.0226	
A²	68.54	1	68.54	64.42	<0.0001	
B²	8.44	1	8.44	7.94	0.0182	
C²	141.2	1	141.2	132.72	<0.0001	
Residual	10.64	10	1.06			
Lack of Fit	10.64	5	2.13	0.0823	0.8823	not significant
Pure Error	162.65	5	51.86			
Cor Total	272.97	19				
R² 0.9861	Model Precision 10.9798					
Adjusted R² 0.9659	Std. Dev.	1.03				
Predicted R² 0.7533	Mean	85.38	C.V.%	1.21

**Table 5:** Analysis of Variance (ANOVA) for Phenol response surface.

Source	Sum of Squares	df	Mean Square	F-value	p-value	
Model	1298.75	9	144.31	5.25	<0.0001	significant
A-Catalyst dosage	5.25	1	5.25	0.1911	0.0413	
B-Run Time	85.43	1	85.43	3.11	0.043	
C-Air Flow rate L/min	315.28	1	315.28	11.48	0.0069	
AB	240.57	1	240.57	8.76	0.0143	
AC	554.24	1	554.24	20.18	0.0012	
BC	94.29	1	94.29	3.43	0.0036	
A²	0.7725	1	0.7725	0.0281	0.0072	
B²	1.74	1	1.74	0.0633	0.0065	
C²	4.65	1	4.65	0.1693	0.0894	
Residual	274.69	10	27.47			
Lack of Fit	22.89	5	4.58	0.0909	0.9901	not significant
Pure Error	251.8	5	50.36			
Cor Total	1573.44	19				
R² 0.9854	Model Precision 9.374					
Adjusted R² 0.9683	Std. Dev.	5.24				
Predicted R² 0.7567	Mean	47.86	C.V.%	10.95		

#### Graphical analysis of individual factors

Graphically, the individual factors studied behaved differently towards the removal of the contaminants, which might be due to the hydrophobic nature of ORW^46^. Since pH (5.5) is one of the major photocatalysis factors, this study was kept constant. It has been reported that in acidic medium (<5.5) the surface charge of TiO_2_ is positive^57,58^. unlike the alkaline medium (>5.5) where it becomes negatively charged^59^. However, most of the organic compounds in ORW (like SOG, phenol, and other phenolic derivatives) are negatively charged^55,56^. Hence acidic medium favors their electrostatic force of attraction towards the TiO_2_ charged surface^59,60^. The Supplementary Fig. S1 presents the effect of the individual factors (A-catalyst dosage; B-reaction time and C- aeration time rate on the two responses (SOG and phenol removal). In both cases, increasing the catalyst dosage as a function of time increased their removal. Whereas, an increase in aeration rate to the maximum with other factors kept constant, resulted in a drop in photocatalytic degradation efficiency^44,53^. Thus at the high aeration rate, most of the radical species might be hunted, hence resulting in reducing the reaction rate and the removal efficiency^59,60^.

#### Interaction effects of factors

**Figure 5:**
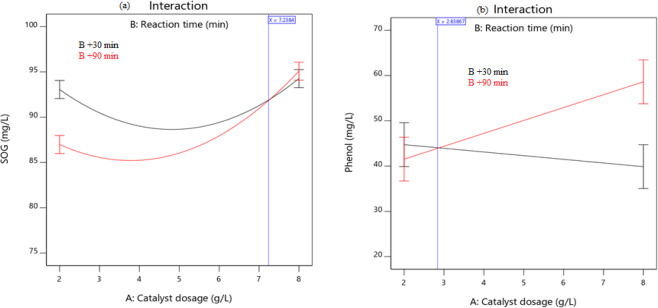
Diagnostic interaction plot of catalyst dosage and reaction time on (**a**) SOG and (**b**) Phenol removal at constant aeration rate (1.2 L/min).

To overcome the setbacks of OFAT techniques (Supplementary Fig. S1), evaluating the interaction effects of the factors were found to be important. Thus, the interaction effects can augment or diminish the main impact on their response^45^. Figure 5 shows the interaction effects of catalyst dosage (A) and reaction time (B) on (a) SOG and (b) phenol removal at fixed aeration of 1.2 L/min. This confirms the ANOVA test, where the two interaction factors (AB) were found to be highly significant (P < 0.0001). The degradation of efficiency was seen to increase with an increase in catalyst dosage with respect to maximum time (B- red line). Whereas with minimum time (B-black line) there was a reduction in performance. Thus, at the maximum dosage, there is a high tendency of agglomeration (particle-particle interaction) with excessive particle concentration at lower reaction rate^50,51^. This might compromise the active surface area available for absorption^58^. The tradeoff between these two opposing phenomena will result in a drop in photocatalytic degradation^59^. Until optimum conditions are attained, there will be non-uniform light intensity distribution within the solution^60^.

**Figure 6:**
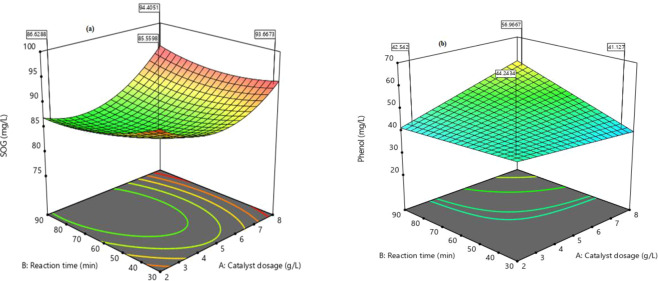
3D response surface plots of the interactive effect of TiO_2_ dosage vs time on (**a**) SOG and (**b**) Phenol removal at a constant aeration rate of 1.2 L/min.

Three-dimensional (3D) plots (Fig. 6) illustrate the relationship between input variables interactions and the response variables (SOG and Phenol removal). The curvatures of the 3D plots clearly show the desirable condition peaks shifted towards the higher catalyst dosage and reaction time^45,61^. Which signifies increasing the catalyst dosage more hydroxyl radicals are produced to enhance the degradation^46,53^. However, TiO_2_ nanoparticles have a high affinity to agglomerate with respect to time, hence the longer the time the better. Thus, aggregation (particle-particle interaction) began at photocatalyst dosage >2.5 g/L, which might reduce the effective surface area of the catalyst and adsorption of the reactants^47,48^.

#### Numerical optimization

**Figure 7:**
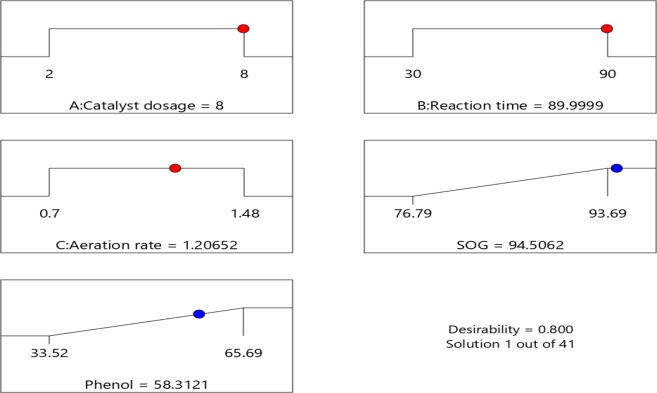
Selected CCD ramp plots of optimized conditions with desirability performance of 80%.

In numerical optimization, the desirability function was carried out for each response and individual factors. The possible goal to maximize the responses were set together with the optimum region of the factors minimum and maximum levels^45^. The desirability values of the numerical optimization procedure was set in terms of catalyst dosage (2–8 mg/L), runtime (30–90 min), and airflow rate (0.768–1.48 L/min). The desirability goal was randomized and more than two maximum goals were attained due to their curvature in the response surfaces and their combination in the desirability function [Design Expert software]. To achieve high desirability goals, about 41 optimized conditions were obtained within the designed space (presented in supplementary Table S1). Figure 7 shows the most suitable option selected with a desirability efficiency of 80% representing 95% SOG and 58% phenol, at a catalyst dosage of 8 mg/L, run time of 90 min, and an airflow rate of 1.225 L/min.

### Confirmation test and kinetic study

**Figure 8:**
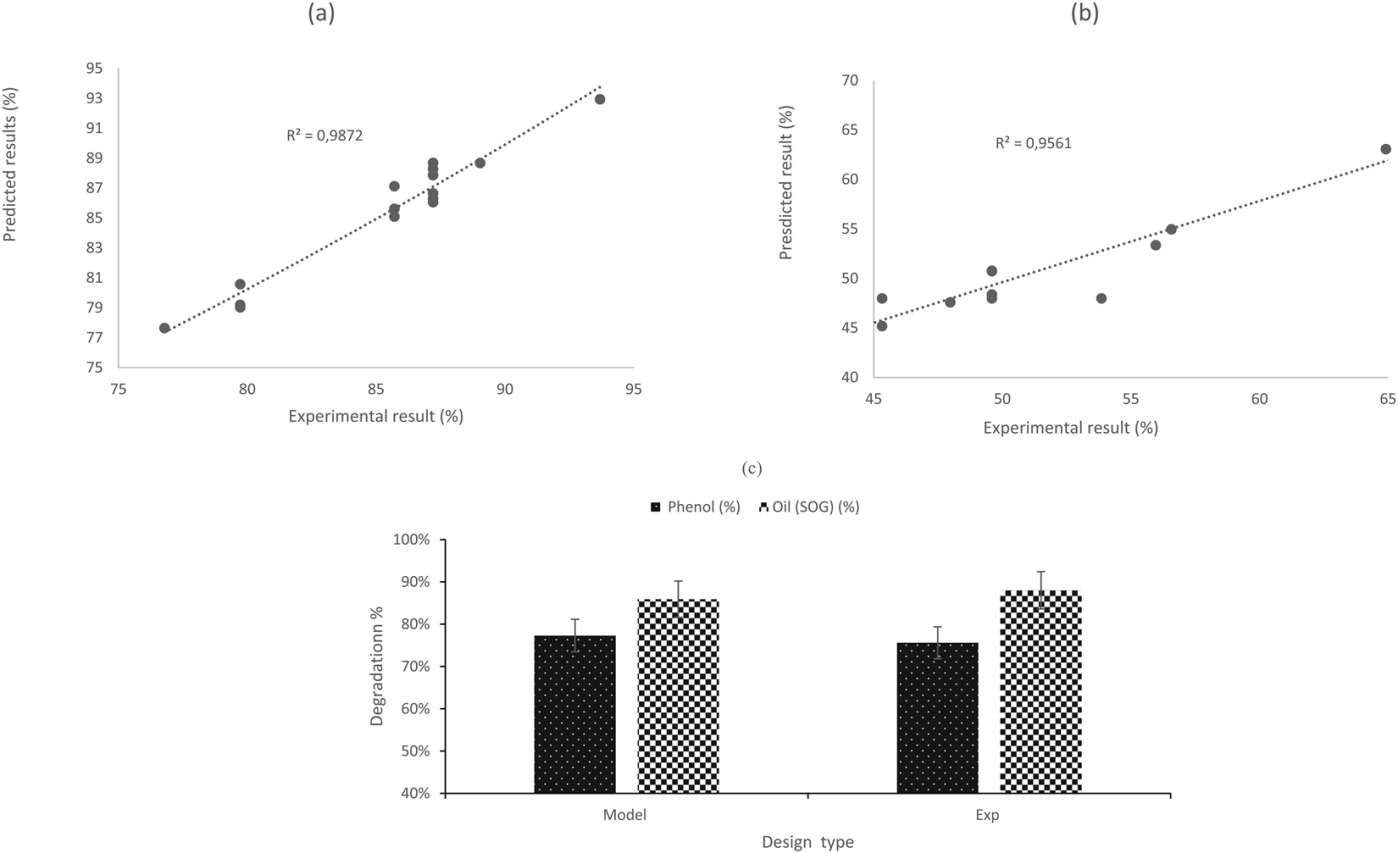
Model validation; predicted vs actual (**a**,**b**) for SOG and phenol removal respectively; (**c**) confirmation test at optimum conditions of catalyst dosage 8 mg/L, runtime 90 min and airflow rate 1.225 L/min.

In Fig. 8, the overall experimental results were seen to be in good agreement with the model predicted results (a and b). The optimum conditions (Fig. 7) were confirmed experimentally with 88% and 76% removal of SOG and Phenol respectively (Fig. 8c). The model developed also predicted 86% and 77% for SOG and phenol removal, with a deviation of less than 5%. The photocatalysis with TiO_2_ (Degussa P25) for the treatment of ORW containing SOG and phenolic products was found to be feasible, where the system efficiency was significantly based on the catalyst dosage as a function of the reaction time. The data obtained was successfully tested with the pseudo-first-order kinetics which showed a linear progression behaviour of degrading ORW contaminants. By using (1), the function of ln (C/Co) vs reaction time (min) was plotted, after which the kinetic rate constant (k) was estimated. Respectively the SOG and Phenol k values obtained were 0.136 min^−1^ and 0.083 min^−1^. The slow kinetic degradation of the phenol might be due to the presence of intermediaries, which occurred during the reaction after 30 min of irradiation. This results in limiting reactive sites and light penetration available per unit volume for the photocatalytic reaction.

## Conclusion

This study demonstrated the feasibility of employing RSM to evaluate the photocatalytic degradation of ORW with TiO_2_ (Degussa P25) photocatalyst is viable. Analysis of variance (ANOVA) test was used to evaluate the individual and interaction effect of the three input factors (catalyst dosage, runtime, and airflow rate) on the photocatalytic degradation performance. The experimental data were well fitted on a second-order model for the response variables (SOG and Phenol) as a function of the input variables. ANOVA results for the quadratic models developed showed high coefficients of determination (R^2^) of 0.9861 and 0.9854 for SOG and Phenol removal respectively and were both significant (P < 0.0001). Additionally, all the model (equations) terms were highly significant, with catalyst dosage being the most influential factor. With the data obtained from the CCD matrix, the ANOVA revealed the increasing order of interaction factors on the response as catalyst dosage and reaction time (AB) > catalyst dosage and aeration rate (AC) > reaction time and aeration rate (BC). Under the optimized conditions of reaction time (90 minutes), TiO_2_ dosage (8 g/L), and aeration rate (1.225 L/min), the system desirability performance of 80% removal of the pollutants (SOG and Phenol) was achieved. The model developed predicted results were validated experimentally under the same optimized conditions. The results obtained were well agreeable with the software predicted results at 95% confidence. Successfully, with the experimental designed data obtained, the first-order kinetic rate constants were determined as 0.136 min^−1^ and 0.083 min^−1^, which respectively described the photocatalytic degradation kinetics of the SOG and Phenol from the ORW. However, the amount of phenol (69 ± 15 mg/L) and SOG (480 ± 36) that were left after the treatment was seen to be greater than their respective discharge limits of 5 mg/L and 50 mg/L. Within the same design space as this study, the photocatalysis process is recommended to be used as a polishing step. Additional modification of TiO_2_ (Degussa P25) is essential to improve its treatability and recoverability.

## Supplementary information

Supplementary information.

Table S1.
