# Supplementary material for: Photocatalytic degradation of oily waste and phenol from a local South Africa oil refinery wastewater using response methodology
Source: Sci Rep. 2020 Jun 1;10:8850. doi: 10.1038/s41598-020-65480-5 (PMC7264190; doi:10.1038/s41598-020-65480-5)
Supplement: Supplementary file 1 — Supplementary information. [file 41598_2020_65480_MOESM1_ESM.docx]

**Photocatalytic degradation of oily waste and phenol from a local South Africa oil refinery wastewater using response methodology**

**E K Tetteh , S Rathilal and DB, Naidoo**

Faculty of Engineering and the Built Environment, Department of Chemical Engineering, Durban University of Technology, Steve Biko Campus Block S4 Level 1, Box 1334, Durban 4000, South Africa.

* Corresponding author email: [ektetteh34@gmail.com](mailto:ektetteh34@gmail.com) ORCID;  [0000-0003-1400-7847](http://orcid.org/0000-0003-1400-7847)


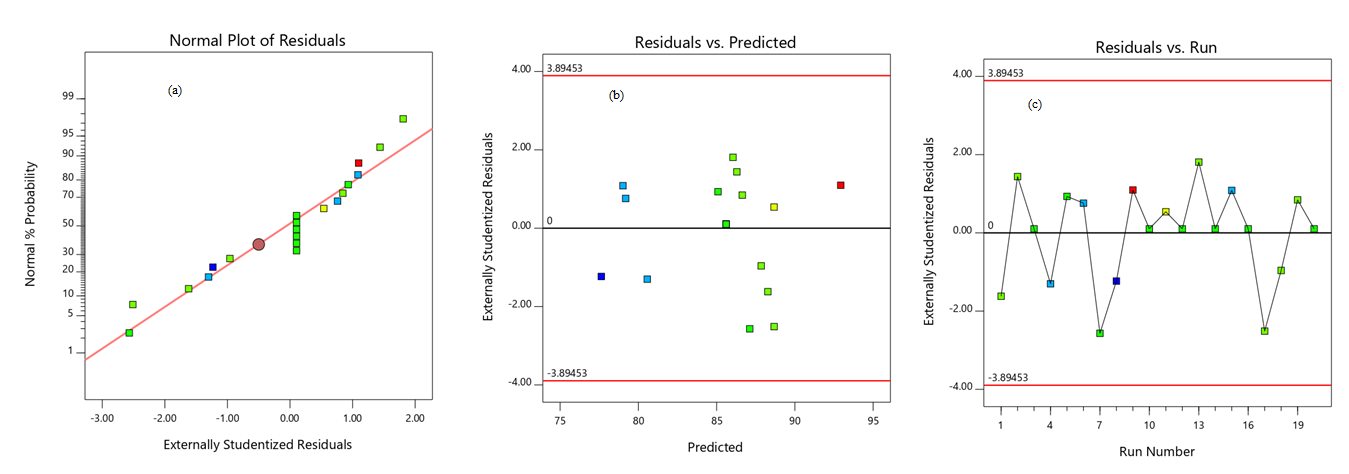


Fig S1 : Diagnostic plots; (a) normal probability ; (b) residual vs predicted ; (c) residuals vs experimental runs . The normal probability of the residuals ( a), to verify if the standard deviation between the actual and the predicted response values corresponse to a normal distribution (b) , the residuals display falls closely to the centered straight line , incadicating there was no abnormality between the predicted and experimental results. All the data points were randomly scattered within the constant range of residuals across the graph ( c), which signifies the models are adequate and precise.


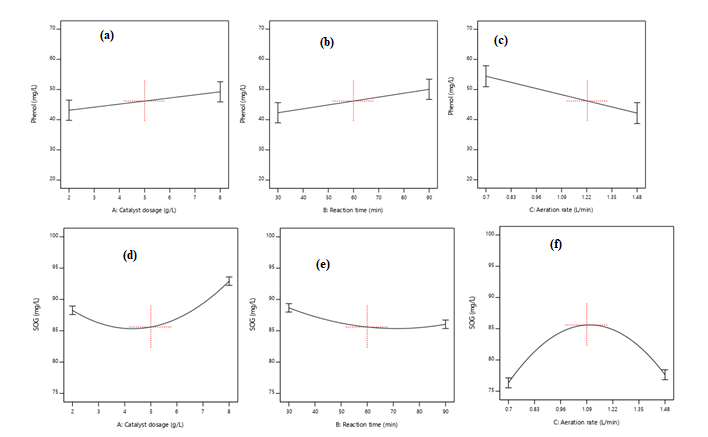


Fig S2 Effects of catalyst dosage, reaction time and aeration rate repectively on phenol removal (a; b; c) and SOG removal (d; e; f). The trends of the factors towards phenol removal (a; b; c) were seen to be linear , whereas that of the SOG removal (d; e; f) were exponential. In both cases, increasing the the catalyst dosage ( a; d) as function of time ( b; e) imcreased their removal. Whereas, an increase in aeration rate (c; f) to maximum decreased the photocatalysis efficiency. Thus at the high aeration rate , most of the radical species might be hunted. Resultant of this reduction of the rate of reaction and a drop photocatalytic degradation efficiency.
